# To Generate an Ensemble Model for Women Thyroid Prediction Using Data Mining Techniques

**DOI:** 10.31557/APJCP.2019.20.4.1275

**Published:** 2019

**Authors:** Dhyan Chandra Yadav, Saurabh Pal

**Affiliations:** *VBS Purvanchal University, Jaunpur, U.P., India. *

**Keywords:** Meta classifier algorithms, boosting, bagging, ensemble-I, ensemble-II, ROC, MAE, RMSE, RAE, RRSE

## Abstract

**Objective:**

The main objective of this paper is to easily identify thyroid symptom for treatment.

**Methods:**

In this paper two main techniques are proposed for mining the hidden pattern in the dataset. Ensemble-I and Ensemble-II both are machine learning techniques. Ensemble-I generated from decision tree, over fitting and neural network and Ensemble-II generated from combinations of Bagging and Boosting techniques. Finally proposed experiment is conducted by Ensemble-I vs. Ensemble-II.

**Results:**

In the entire experimental setup find an ensemble –II generated model is the higher compare to other ensemble-I model. In each experiment observe and compare the value of all the performance of ROC, MAE, RMSE, RAE and RRSE. Stacking (ensemble-I) ensemble model estimate the weights for input with output model by thyroid dataset. After the measurement find out the results ROC=(98.80), MAE= (0.89), 6RMSE=(0.21), RAE= (52.78), RRSE=(83.71)and in the ensemble-II observe thyroid dataset and measure all performance of the model ROC=(98.79), MAE= (0.31), RMSE=(0.05) and RAE= (35.89) and RRSE=(52.67). Finally concluded that (Bagging+ Boosting) ensemble-II model is the best compare to other.

## Introduction

It is very critical to observe and measure combination of hormonal disturbance in women. It is not detected only in ladies but also find in gens. The reason behind thyroid is flexuation of hormones over or low in the human. It is very necessary for healthcare to balance hormonal over or low variation of hormones’. Hormonal disturbance have some risk factors so it is more required to continuous concern for the doctors and find the correct diagnosis at the correct time. Some very importance questions in thyroid to making decision as like what is most important technique to classify and identify thyroid symptoms? How treats in this situation? How minimize the thyroid symptom? How take best decision to minimize death risk? In this paper focus their work and using different ensemble models to identify the best algorithms for classification thyroid disorders. Thyroid glands have the shape as like butterfly. Thyroid gland produces two different type hormones a like T3 and T4. These hormones manage the effective function of body as like body temperature, blood pressure, heart rate sexual system etc. In some happening if T3 and T4 are not in proper way then some difference problems arise as like Hyperthyroidism and Hypothyroidism. If T3 is high and T4 normal means thyroid gland produce much hormone it will be Hyperthyroid and in other hand if T3 is less and T4 is normal then it will be Hypothyroid. This paper is organized in the two different sections. Data mining technique in healthcare observed all the discovering patterns between various collections of thyroid dataset for women (Farwell, 2019).

Diagnosis of thyroid disease in which the thyroid gland produces hormones to maintain metabolism of the human body. The thyroid disorders are classified into three parts first is Hypothyroidism second is the Hypothyroidism and third is Euthyroidism. In this paper author used machine learning methods linear discriminate analysis, K-nearest neighbour and adaptive neuro-fuzzy inference system. In the final analysis author find out accuracy (98.5%), sensitivity (94.7%) and specificity of this approach (Ahmad et al., 2018). A combine method of adaptive neuro-fuzzy inference system and information gain method. They decreased computation time and classification complexity. They find out classification accuracy (95.24%), specificity (91.7%) and sensitivity (96.17%) (Ahmad et al., 2018). A system by machine learning method for thyroid disease and the effectiveness of analyzed system measured in term of classification accuracy (Ma et al.,2018). Classification tree and its accuracy (98.89%) over the other classification techniques. They used k-nearest neighbours, support vector machine, decision tree and naïve bayes (Umar Sidiq et al., 2019).

Thyroid disease are analyzed by J48 graft and they take (7,200) thyroid dataset due to hypothyroidism and hyperthyroidism. They measure maximum classification accuracy (97.02%) and they suggested a model for thyroid disease (Hayashi, 2017). Thyroid prediction for women by test hyperthyroidism and hypothyroidism. They supported as a mini expert for dysfunction. They provided best clinical result comparison to traditional clinical practices (Kusić et al., 2009). Thyroid cancer by classification technique are discussed to analyzed thyroid cancer by digital image of the cell. They used template machine technique and automated defect and improve the accuracy of classification with very short time (Jagdeeshkannan et al., 2014). The disease used classification, Data mining, Decision tree and thyroid diagnosis for the prediction of thyroid disease. They performed and measure six metrics Accuracy, roid MAE, PRE, REC, FME and Kappa statistic and finally they find out NB tree analyzed (75%) highest rate accuracy (Turanoglu-Bekar et al., 2016). Thyroid disease by machine learning algorithms and they used machine learning, thyroid disease CRT decision tree and python for best knowledge in medical science. Finally they find out machine learning tool for thyroid disease diagnosis with 99.7% accuracy (Al-muwaffaq and Bozkus, 2016). Authors discussed about thyroid disease four classification model of data mining. They used data mining classification model, thyroid disease, neural network, decision tree and naïve bayes for dysfunctions among the population and detect more effect of thyroid on women. They also find out all the performance of decision tree, clustering and all selected algorithms performance high accuracy and efficiency (Priyal and Anitha, 2017). 

Thyroid organ and hormones productions are discussed and they used hormones, hypothyroidism and risk prediction for controlling the body digestion. They find out in the thyroid prediction system. Naïve bayes predict with hypothyroid and find out the best outcomes for accuracy and least execution time (Vijaylakshmi et al., 2018). Paper discusses about thyroid challenging factors. They used thyroid disease, decision tree, naïve bayes, SVM and Backpropogation. They find out various results on speed, accuracy, performance and cost in prediction of thyroid dataset. These techniques minimize the noisy data of the thyroid patient data (Rajam et al., 2016). Role of machine learning in medical science by the help of Meta classifier. They used data mining decision tree and regression tree in multiple ways. They find 93% classification accuracy and suggested to boost algorithm for prediction (Chaurasia et al., 2018). Machine learning used data quality, data repairing, data inconsistency and data cleaning for quality of data. They conducted the study for effectiveness comparison of existing miner techniques. They observed the response time, storage space and database scability (Salem and Abdo, 2016). Authors discussed about efficient and effective method for feature subset selection. They used classification, evolutionary computation techniques, decision tree and nearest neighbour for higher classification accuracy. They observed both generic algorithms, RST based algorithm and increased accuracy of the classification (Surekha and Suma, 2016). This paper discussed breast treatment of cancer for women. They used Breast, Data Mining, Naïve Bayes and RBF Network. They predicted the breast cancer by many classifier algorithms and Naïve Bayes give the highest accuracy (97.36%) (Chaurasia et al., 2015). Authors discussed about anti- thyroid peroxides for classification. They used T3, T4 and TSH associative increase risk of birth and miscarriage for defection hyperthyroidism and hypothyroidism (Loh et al., 2016).

## Materials and Methods

All the dataset used for experimental and study purpose. All the observation is dividing in following section: data description, algorithm description, result discussion and then find conclusion. 

*Data Description*

All the dataset collect from some different source as like Rahul pathology, Chandan pathology and some data collect from website. All the dataset divide into three parts: First is 5,000 thyroid dataset instances second is 10,000 and third is 12,000 instances. The dataset categories some negative and some positive category. The target variable class level divide into three parts (1) Hyperthyroid, (2) Hypothyroidism and (3) Euthyroidism but finally observe and predict hypothyroidism class (Farwell, 2019). All the related details of thyroid dataset are presented in table -1.

**Table 1:** Computational Table for Thyroid Dataset Variables

Source	Chandan Diagonosis Center, Rahul pathology and Rahul thyroid diagnosis center,
	https://github.com/mikeizbicki/datasets/blob/master/csv/uci/new-thyroid.names
Sample Size	Total:12,000, Hypothyroidism: 5,628, Hyperthyroidism: 5,522, Euthyroid State:0850
Dependable Variables	
Observation (Low)	Hypothyroidism in which a person’s hormone production is below normal.
Observation (High)	Hyperthyroidism in which a person’s thyroid over produces hormones.
Observation (Normal)	Euthyroid State is a normal thyroid gland function.
Independable Variables	
Fatigue (tired)	1= H, 0= L and 2= Ir.
Cold Intolerance	1= R, 0= N and 2= Ir.
Skin	0= LessDry , 1= Dry and 2=Normal.
Weight	0=Loss, 1= Gain.
Face Swelling	1= Detect,2 = Normal.
Menstrual Cycles	0=Infrequent& Less Bleeding, 1= Early & High Bleeding, 2=Normal
Hair	1=Loss, 2=Normal.
Memory Concentrating	1=Weak and 2=Normal.
Heart Rate	1=High, 2= Normal and 0=Slow.
Bowel Movements	2=Normal, 1=Frequent and 0=No Frequent.
Hand Tremors	1=Stronger, 2=Normal and 0= Weak.
Blood Pressure	1=High, 0=Low and 2=Normal.

**Table 2:** Computational Table of 5,000 Instances for Thyroid Dataset

Algorithms	ROC	Mean absolute error	Root mean squared error	Relative absolute error	Root relative squared error	No. of Instances
Bagging	98.52	0.85	0.06	23.34	40.61	
AdaBoostinhM1	95.39	0.91	0.08	37.65	52.29	
Ensemble-I (Stacking)	96.89	0.79	0.05	29.52	39.64	5000
Ensemble-II (Bagging+ Boosting)	98.35	0.81	0.03	19.75	32.74	

**Table 3:** Computational Table of 10,000 Instances for Thyroid Dataset

Algorithms	ROC	Mean absolute error	Root mean squared error	Relative absolute error	Root relative squared error	No. of Instances
Bagging	97.78	0.72	0.12	46.67	71.64	10000
AdaBoostinhM1	98.92	0.53	0.09	39.42	53.69	
Ensemble-I(Stacking)	96.8	0.89	0.21	52.78	83.71	
Ensemble-II (Bagging+ Boosting)	98.45	0.49	0.07	37.83	51.93	

**Table 4:** Computational Table of 12,000 Instances for Thyroid Dataset

Algorithms	ROC	Mean absolute error	Root mean squared error	Relative absolute error	Root relative squared error	No. of Instances
Bagging	95.98	0.79	0.23	72.31	98.47	12,000
AdaBoostinhM1	96.39	0.67	0.19	69.72	95.38	
Ensemble-I (Stacking)	97.1	0.53	0.13	59.72	98.51	
Ensemble-II (Bagging+ Boosting)	98.79	0.31	0.05	35.89	52.67	

**Figure 1:**
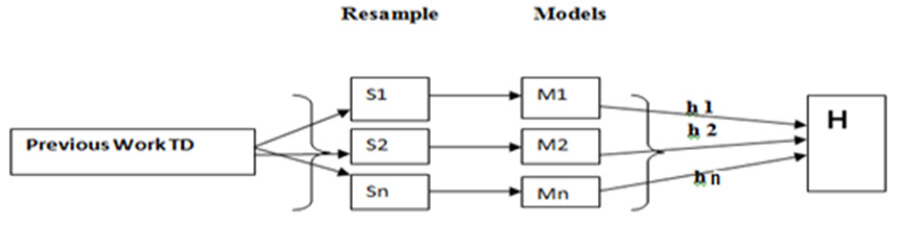
Uniform Re-Sample Modelling of Thyroid dataset in Parallel Style by Bagging Algorithm

**Figure 2:**
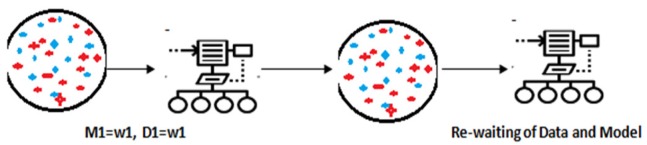
Reweighting with Non-Sequential Modelling of Thyroid Dataset by Boosting Algorithm

**Figure 3:**
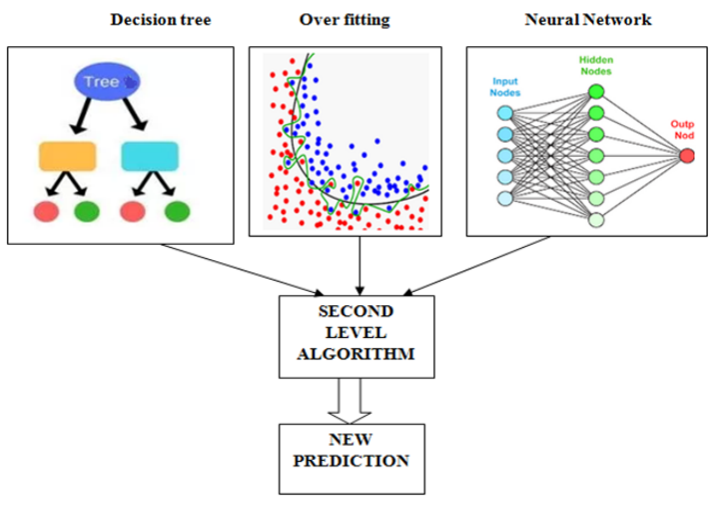
Observation of Thyroid Dataset by Stacking Algorithm

**Figure 4:**
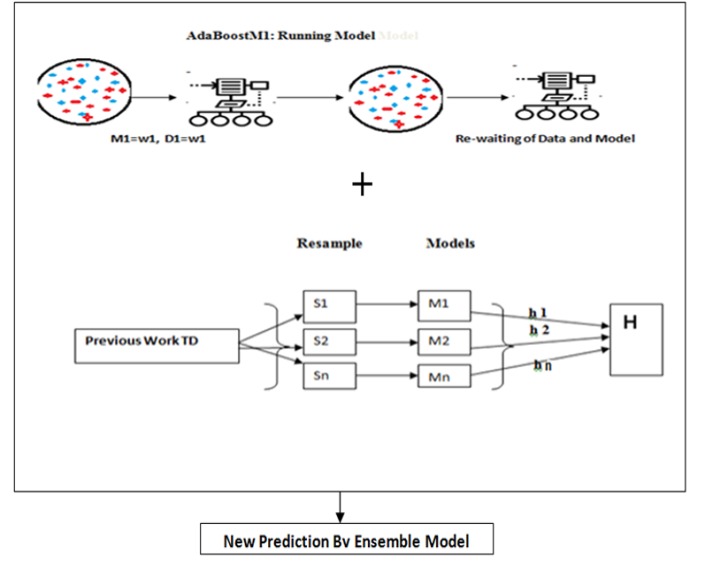
Propose Ensemble Model for Prediction of Thyroid Dataset

**Figure 5:**
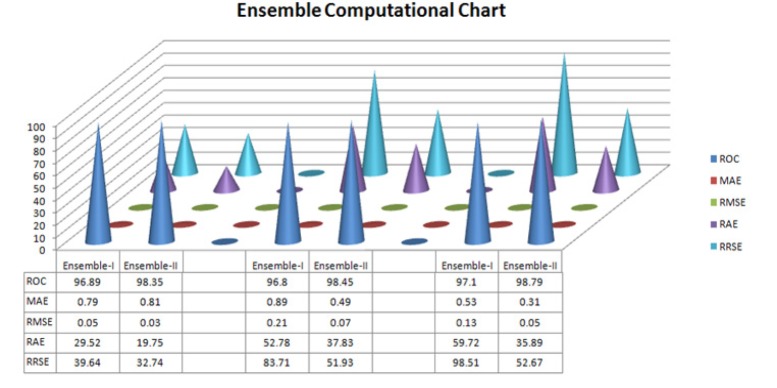
Computational Figure for Ensemble-I vs. Ensemble-II of Thyroid Dataset

In this paper observe the prediction in by the low, high and normal dependable variable. Low high and normal dependable variable have his class level state mention in above table -1. 

Some other in-dependable Variables: Fatigue (tired), Cold Intolerance, Skin, Weight, Face Swelling, Menstrual Cycles, Hair, Memory Concentrating, Heart Rate, Bowel Movements, Hand Tremors, and Blood Pressure. All In-dependable variables have his definition and concept mention in the above Table 1. 

*Algorithms Description*

The propose model is not a doctor but it is assist the doctor. Analyst takes support by ensemble model after the collection of all symptoms of thyroid data. In this paper select three Meta classifier algorithms: Bagging, Boosting and Stacking. We discuss about these algorithms as below:

Bagging: Data mining classifier has Meta classifier for prediction. In this research paper bagging algorithm decrease the variance of prediction by formulating thyroid dataset. In the example select previous work training data and then select these data resample S1, S2..Sn. After that formatted as in different models M1, M2...Mn. Finally find the function H from all different modelled functions of h1, h2 …hn (Brownlee, 2019 ).

Boosting: Boosting is machine learning algorithm. Boosting algorithm produce a series of average performing model by subset of original data.Boosting behaves as like a tracker for the data sample with heavier weights. In this paper use M1, W1 and D1 for data model, weights of data model and data sample for training respectively (Brownlee, 2019).

*Stacking as Ensemble Model*

Stacking is a machine learning algorithm. In this paper stacking use as a ensemble model by decision tree, over fitting and neural network. The ensemble model estimates the weights for input with output model. The second layer is train and consist all over three algorithms predictions and also generate new trend for predictions (Brownlee, 2019).

*Proposed model*

In this paper propose ensemble generate in three stages. In the first stage model-I generate from decision tree, over fitting and neural network. These three different algorithms easily evaluate all the features of the thyroid dataset of women in maximum relative direction. I n the second stage generates ensemble model-II from bagging and boosting algorithms. These two different algorithms generate a model for thyroid dataset of women with all relative features of algorithms. In the stage-III easily compare the performance of ensemble-I and ensemble-II and finally evaluate ROC, MAE, RMSE, RAE and RRSE.

Analyse thyroid dataset of women in various way by machine learning. Both ensemble models generate different values in the experiment. In this paper measure ROC, MAE, RMSE, RAE and RRSE.

All the experiment divides in three stages:

## Results

After the various experimental setups we find the result in various ways to describe the classification ROC, MAE, RMSE, RAE and RRSE measure as describe below-

*Experiment-I*

In the first experiment used 10 fold cross validation with (60%) percentage supply. Firstly observe Bagging algorithm and decrease the variance of prediction by thyroid dataset and measure all the performance of ROC, MAE, RMSE, RAE and RRSE. After the measurement find the results ROC=98.52, MAE= 0.85, RMSE=0.06 and RAE=23.34 and RRSE=40.61. AdaBoostM1 algorithm produces a series of average performing model by subset of original data by thyroid dataset and measure all the performance of ROC, MAE, RMSE, RAE and RRSE. After the measurement find the results ROC=95.39, MAE= 0.91, RMSE=0.08 37.65 and RAE= 37.65 and RRSE=52.29.

Stacking(ensemble-I) algorithm The ensemble model estimate the weights for input with output model by thyroid dataset and measure all the performance of ROC, MAE,RMSE,RAE and RRSE. After the measurement find the results ROC=96.89, MAE= 0.79, 6RMSE=0.05 and RAE= 29.52 and RRSE=39.64.

By ensemble-II observe thyroid dataset and measure all the performance of ROC, MAE, RMSE, RAE and RRSE.

After the measurement find the results ROC=98.35, MAE= 0.81, RMSE=0.03 and RAE= 19.75 and RRSE=32.74.

*Experiment-II*

In the second experiment used 10 fold cross validation with (60%) percentage supply. Firstly observe Bagging algorithm and decrease the variance of prediction by thyroid dataset and measure all the performance of ROC, MAE, RMSE, RAE and RRSE. After the measurement find the results ROC=97.78, MAE= 0.72, RMSE=0.12 and RAE=46.67 and RRSE=71.64.

AdaBoostM1 algorithm produce a series of average performing model by subset of original data by thyroid dataset and measure all the performance of ROC, MAE, RMSE, RAE and RRSE. After the measurement find the results ROC=98.92, MAE= 0.53, RMSE=0.09 and RAE= 39.42 and RRSE=53.69.

Stacking(ensemble-I) algorithm The ensemble model estimate the weights for input with output model by thyroid dataset and measure all the performance of ROC, MAE,RMSE,RAE and RRSE. After the measurement find the results ROC=98.80, MAE= 0.89, 6RMSE=0.21 and RAE= 52.78 and RRSE=83.71.

By ensemble-II observe thyroid dataset and measure all the performance of ROC, MAE,RMSE,RAE and RRSE.

After the measurement find the results ROC=98.45, MAE= 0.49, RMSE=0.07 and RAE= 37.83 and RRSE=51.93.

*Experiment-III*

In the Third experiment used 10 fold cross validation with (60%) percentage supply. Firstly observe Bagging algorithm and decrease the variance of prediction by thyroid dataset and measure all the performance of ROC, MAE, RMSE, RAE and RRSE. After the measurement find the results ROC=95.98, MAE= 0.79, RMSE=0.23 and RAE=72.31 and RRSE=98.47.

AdaBoostM1 algorithm produce a series of average performing model by subset of original data by thyroid dataset and measure all the performance of ROC, MAE, RMSE, RAE and RRSE. After the measurement find the results ROC=96.39, MAE= 0.67, RMSE=0.19 and RAE= 69.72 and RRSE=95.38.

Stacking (ensemble-I) algorithm The ensemble model estimate the weights for input with output model by thyroid dataset and measure all the performance of ROC, MAE,RMSE,RAE and RRSE. After the measurement find the results ROC=97.10, MAE= 0.53, 6RMSE=0.13 and RAE= 59.72 and RRSE=98.51.

By ensemble-II observe thyroid dataset and measure all the performance of ROC, MAE,RMSE,RAE and RRSE.

After the measurement find the results ROC=98.79, MAE= 0.31, RMSE=0.05 and RAE= 35.89 and RRSE=52.67.

Finally find out in all the observation Ensemble-II generate model give high ROC compare to other ensemble-I algorithm set model and give less error values compare to all other error MAE,RMSE,RAE and RRSE.

In conclusion, in all the experiment used 10 fold cross validation with (60%) percentage supply. Experiment-I observe thyroid dataset for 5,000 instances and measure all the performance of ROC, MAE,RMSE,RAE and RRSE. After the measurement find the results ROC=98.35, MAE= 0.81, RMSE=0.03 and RAE= 19.75 and RRSE=32.74 .The performance of Ensemble-II is higher compare to Bagging, AdaboostM1 and Ensemble-I.

Experiment-II observe thyroid dataset for 10,000 instances and measure all the performance of ROC, MAE,RMSE,RAE and RRSE. After the measurement find the results ROC=98.45, MAE= 0.49, RMSE=0.07 and RAE= 37.83 and RRSE=51.93. The performance of Ensemble-II is higher compare to Bagging, AdaboostM1 and Ensemble-I.

Experiment-II observe thyroid dataset for 12,000 instances and measure all the performance of ROC, MAE,RMSE,RAE and RRSE. After the measurement find the results ROC=98.79, MAE= 0.31, RMSE=0.05 and RAE= 35.89 and RRSE=52.67. The performance of Ensemble-II is higher compare to Bagging, AdaboostM1 and Ensemble-I.

## Discussion

In all the experimental setup find an ensemble –II generated model is the higher compare to other ensemble-I model. In each experiment observe and compare the value of all the performance of ROC, MAE, RMSE, RAE and RRSE. Stacking (ensemble-I) algorithm The ensemble model estimate the weights for input with output model by thyroid dataset . After the measurement find out the results ROC=98.80, MAE= 0.89, 6RMSE=0.21, RAE= 52.78, RRSE=83.71and in the ensemble-II observe thyroid dataset and measure all performance of the model ROC=98.79, MAE= 0.31, RMSE=0.05 and RAE= 35.89 and RRSE=52.67. Finally concluded that (Bagging+ Boosting) ensemble-II model is the best compare to other. For future research some ensemble model with different computational technique and decision tree can be used for clustering and association of the thyroid disease.

## Statement conflict of Interest

The authors declare no conflict of interest.
